# Physiological responses of three species of unionid mussels to intermittent exposure to elevated carbon dioxide

**DOI:** 10.1093/conphys/cow066

**Published:** 2016-12-29

**Authors:** Kelly D Hannan, Jennifer D Jeffrey, Caleb T Hasler, Cory D Suski

**Affiliations:** Department of Natural Resources and Environmental Science, University of Illinois at Urbana-Champaign, 1102 South Goodwin Avenue, Urbana, IL 61801, USA

**Keywords:** Acid–base regulation, bivalve, freshwater acidification, ions

## Abstract

Freshwater systems are at risk owing to increasing carbon dioxide (CO_2_) levels, and one of the possible reasons for these elevations is the deployment of non-physical fish barriers to prevent invasive fish movements. Carbon dioxide barriers have the potential to create short, chronic and intermittent exposures of CO_2_ for surrounding freshwater biota. Although intermittent exposures to a stressor may be more ecologically relevant, the majority of laboratory tests use chronic or short-term time periods to determine how organisms will respond to an environmental stressor. Measurements of the physiological responses of three species of unionid mussel, giant floaters (*Pyganodon grandis*), threeridge (*Amblema plicata*) and plain pocketbook (*Lampsilis cardium*), exposed to control *p*CO_2_ (~1000 µatm) or intermittent conditions of *p*CO_2_ (ranging from ~1000 to ~55 000 µatm) 12 times per day over a 28 day period were gathered. There was no indication of recovery in the physiological responses of mussels between applications of CO_2_, suggesting that the recovery time between CO_2_ pulses (1.5 h) was not sufficient for recovery from the CO_2_ exposure period (0.5 h). Observations of acid–base and stress responses were consistent with what has been observed in chronic studies of freshwater mussels exposed to elevated *p*CO_2_ (i.e. elevations in HCO_3_^−^, Ca^2+^, Na^+^ and glucose, and decreases in Mg^2+^ and Cl^−^). However, species differences were observed across almost all variables measured, which emphasizes the need for multispecies studies.

## Teaser

Zones of elevated carbon dioxide (CO_2_) have been proposed as barriers to fish movements, but impacts of CO_2_ on non-target sensitive organisms are unknown. In the present study, freshwater mussels were exposed to intermittent elevations in CO_2_ and showed species-specific mechanisms to regulate acid–base disturbances, along with a stress response.

## Introduction

Environmental levels of carbon dioxide (CO_2_) that are commonly found in freshwater ecosystems have the potential to act as both continuous and intermittent stressors for aquatic organisms. Over the past several decades, levels of CO_2_ in the atmosphere have been increasing as a result of the anthropogenic burning of fossil fuels, which has led to a concomitant increase in the partial pressure of CO_2_ gas (*p*CO_2_) in marine ecosystems (Shirayama and Thornton, 2005). Unlike marine systems, there is no consensus regarding how *p*CO_2_ will change in freshwater as a result of climate change (Hasler *et al*., 2016). In freshwater, *p*CO_2_ can vary across and within watersheds (Butman and Raymond, 2011), as well as episodically and on seasonal and diel cycles within water bodies (Maberly, 1996). In a review of ~7000 global rivers and streams, the average median value for *p*CO_2_ was ~3100 µatm (Raymond *et al*., 2013), and in another global review of 47 large rivers the means varied from 679 ± 543 to 35 617 ± 46 757 µatm, with means in the USA ranging from 679 ± 543 to 9475 ± 993 µatm (Cole and Caraco, 2001). In addition to these natural sources of elevated *p*CO_2_, recent work has shown that zones of elevated CO_2_ can act as non-physical fish barriers, thereby providing a management tool to prevent the movement and spread of invasive fish species (Kates *et al*., 2012; Noatch and Suski, 2012). Although a specific method for the use of CO_2_ barriers to deter fish movement has not yet been defined, one potential application is the intermittent addition of CO_2_ into a navigational lock or approach channel at vulnerable times (i.e. when lock doors are open; United States Army Corps of Engineers, 2014a), resulting in downstream pulses of CO_2_-rich water. Thus, downstream fluctuations in CO_2_ might occur, making CO_2_ a potential intermittent stressor for freshwater organisms.

A taxonomic group of freshwater organisms that may be particularly at risk to CO_2_ stressors are freshwater mussels (Order Unionoida). Mussels serve many important ecological functions, influence many ecosystem processes (Vaughn and Hakenkamp, 2001) and are often used as indicators of ecosystem health (Williams *et al*., 1993). Although North American freshwater ecosystems contain the highest diversity of freshwater mussels in the world (Williams *et al*., 1993; Bogan, 2008), more than half (71%) are listed as endangered, threatened or of special concern, largely as a result of anthropogenic stressors, such at habitat alteration and degradation (Williams *et al*., 1993; Ricciardi *et al*., 1998). Additionally, while mussels are generally considered a homogeneous group of sessile animals, there are four main tribes of mussels in North America (Quadrulini, Lampsilini, Pleurobemini and Amblemini) that all vary in morphology, physiology and reproductive strategies and may thus respond differently to environmental stressors.

At present, there is a paucity of research on the effects of elevated *p*CO_2_ on freshwater invertebrates, particularly unionid mussels. Hannan *et al*. (2016a, b) found that mussels experience acid–base regulation in response to short- and long-term exposures to elevated *p*CO_2_, and a stress response to long-term exposure to elevated *p*CO_2_. Previous studies on marine bivalves indicate that elevated *p*CO_2_ causes internal acidosis (Michaelidis *et al*., 2005; Bibby *et al*., 2008) that is often buffered by increasing HCO_3_^−^ in the fluids (Pörtner *et al*., 2004). Both marine (Michaelidis *et al*., 2005) and freshwater mussels (Hannan *et al*., 2016a, b) can increase haemolymph HCO_3_^−^ by using CaCO_3_ released from the shell as a result of decreased pH and elevated CO_2_ (i.e. increases both haemolymph HCO_3_^−^ and Ca^2+^) or by reducing the activity of the Cl^−^–HCO_3_^−^ exchanger to retain HCO_3_^−^ at the cost of Cl^−^ uptake (Byrne and Dietz, 1997; Hannan *et al*. 2016a, b). Another strategy to buffer acidosis is to alter the activity of Na^+^–H^+^ exchangers to increase removal of H^+^ ions, thus also increasing Na^+^ uptake (Byrne and Dietz, 1997; Lannig *et al*., 2010, Hannan *et al*. 2016b). Exposure to a chronic elevation in *p*CO_2_ also appears to initiate the general stress response in mussels, because a decrease in haemolymph Mg^2+^ and an increase in haemolymph glucose have been observed in unionid mussels (Hannan *et al*. 2016a, b). More importantly, previous studies (i.e. studies described above) that have quantified CO_2_ stressors in mussels have used a continuous application of CO_2_ rather than one that was intermittent as might be expected downstream of a CO_2_ barrier, and differences may exist between the continuous application of a stressor relative to one applied intermittently (exacerbation, attenuation or no change; Reinert *et al*., 2002).

Based on this background, the goal of the present study was to quantify the physiological impacts of intermittent exposures to elevated *p*CO_2_ on three species of freshwater mussels each belonging to a different tribe, *Pyganodon grandis* (tribe Anodontini), *Amblema plicata* (tribe Amblemini) and *Lampsilis cardium* (tribe Lampsilini). To accomplish this goal, over a 28 day period the mussels were exposed to either control *p*CO_2_ or intermittent increases in *p*CO_2_ and then sampled for a suite of physiological parameters related to acid–base status and physiological stress. The results of this study help to clarify further how different exposures to elevated *p*CO_2_ affect the acid–base and stress responses of various freshwater mussel species in habitats where *p*CO_2_ fluctuates.

## Materials and methods

### Mussel collection and husbandry

Plain pocketbook (*L. cardium*) and threeridge mussels (*A. plicata*) were collected by benthic grab from the Mississippi River, Cordova, IL, USA, in July 2015. Giant floater mussels (*P. grandis*) were collected by benthic grab from a barrow pit near Champaign, IL, USA, in August 2015. Mussels were taken to the Aquatic Research Facility at the University of Illinois, Champaign-Urbana, IL, USA in coolers (travel time <3 h for *L. cardium* and *A. plicata* and <1 h for *P. grandis*). Upon arrival at the Aquatic Research Facility, all mussels were cleaned of epibionts and tagged for individual identification with a permanent marker (Neves and Moyer, 1988). Once tagged, mussels were placed in three tubs (1136 litres) supplied with water from a 0.04 ha natural, earthen-bottom pond, where they remained for at least 1 week to recover from transport stressors and to acclimate to laboratory conditions (Dietz, 1974; Horohov *et al*., 1992; Dietz *et al*., 1994). All tubs were equipped with a Teco 500 aquarium chiller (TECO-US, Aquarium Specialty, Columbia, SC, USA) and a low-pressure air blower (Sweetwater, SL24H Pentair, Apopka, FL, USA) to maintain aeration. Fifty per cent water changes using pond water were performed weekly to maintain water quality. Mussels were fed a commercial shellfish diet of the following consituents: *Nannochloropsis* sp. 1–2 µm and a mixed diet of *Isochrysis*, *Pavlova*, *Thalassiosira* and *Tertraselmis* spp. 5–12 µm (Instant Algae, Reed Mariculture Inc., Campbell, CA, USA) every other day (American Society of Testing and Materials, 2006; Wang *et al*., 2007), although mussels did not receive supplemental food for 24 h prior to sampling. Temperature and dissolved oxygen (DO) were recorded daily across all holding tanks with a portable meter (YSI 550A, Yellow Springs Instruments, Irvine, CA, USA) and averaged 22°C (21.7 ± 0.1°C, mean ± SEM) and 7.50 mg l^−1^ (7.60 ± 0.06 mg l^−1^). Water pH was measured using a handheld meter (WTW pH 3310 meter, Germany) that was calibrated regularly, and averaged 8.55 ± 0.01 throughout the acclimation period. Dissolved CO_2_ and total alkalinity (TA) concentrations were measured using digital titration kits and averaged 4.86 ± 0.04 mg l^−1^ and 1093.0 ± 27.0 µmol kg^−1^, respectively (Hach Company, Loveland, CO, USA; Titrator model 16,900 catalogue no. 2272700 and catalogue no. 2271900 for CO_2_ and TA, respectively).

### Fluctuating CO_2_ exposure

To define the impacts of fluctuating CO_2_ on mussel physiology, mussels (*L. cardium*, *A. plicata* and *P. grandis*; *n* = 28) were separated into two recirculating treatment systems (92 litres), each with nine 5 litre tanks (adapted from Hohn and Petrie-Hanson, 2007). Systems were maintained as stated above with the exception that one system received a CO_2_ treatment. In the CO_2_ treatment system, *p*CO_2_ was turned on every 1.5 h, and increased from ambient (~1000 µatm, 1355 ± 119 µatm; pH = 7.85 ± 0.02) to ~55 000 µatm (56 492 ± 1342 µatm; pH = 6.62 ± 0.03) by bubbling CO_2_ gas into the water through an air stone (see Supplementary material, Fig. S1). Elevated *p*CO_2_ was held constant at ~55 000 µatm for 0.5 h, for a total of 12 fluctuations per day. Thus, animals were held at elevated *p*CO_2_ levels for 0.5 h and returned to control levels during the 1.5 h recovery period and then raised back up to elevated conditions for 0.5 h, repeatedly during the course of the experiment. A level of 55 000 µatm was targeted because this level has previously been defined as being a potential target CO_2_ level that could deter the movement of fishes (Donaldson *et al*., 2016) and will possibly be the target level of a CO_2_ barrier. Twelve fluctuations per day represents the historical lock usage of Brandon Road Lock (41.5054**°**N, 88.0996**°**W), a possible site for deployment of a CO_2_ barrier within the Des Plaines River, IL, USA (United States Army Corps of Engineers, 2014a, 2015). The target *p*CO_2_ was maintained with a pH controller (PINPOINT^®^, American Marine Inc., CT, USA) that automatically bubbled CO_2_ into the tank system through an air stone should the pH rise above a target level during exposure (Reynaud *et al*., 2003; Riebesell *et al*., 2010). The level of CO_2_ was then returned to ~1000 µatm by bubbling in air though an air stone to off-gas excess CO_2_. An identical recirculating system was used as a control, and mussels in this control system were treated in the same way as animals receiving CO_2_, except that infused CO_2_ gas was replaced with compressed air such that mussels were held continuously at ambient ~1000 µatm (876 ± 108 µatm; pH = 8.13 ± 0.02) *p*CO_2_. A digital timer (DT620 Heavy Duty Digital Timer, Intermatic Inc Spring Grove, IL, USA) was used to control additions of CO_2_ and air. A modified infrared probe was used to measure *p*CO_2_ (Vaisala GMP220 and GMT221, Vantaa, Finland; Johnson *et al*., 2010), along with a CO_2_ titration kit to determine the concentration of CO_2_ (Hach Company, catalogue no. 2272700, Loveland, CO, USA). Before and after the 12.00 h exposure, temperature (21.7 ± 0.1°C) and DO (7.60 ± 0.07 mg l^−1^) were measured as stated above, and the temperature, pH (see above) and TA (2566.3 ± 252.9 µmol kg^−1^) were entered into CO2calc to verify *p*CO_2_ (Robbins *et al*., 2010).

Individual mussels were non-lethally and repeatedly sampled for haemolymph on day 1, 4, 7, 14, 21 or 28 of exposure to fluctuating *p*CO_2_ or control conditions. Mussels were sampled during the 1.5 h period when CO_2_ was at ambient levels, not during the 0.5 h when CO_2_ levels were elevated. Prior to starting this study, it was not known whether sampling mussels immediately prior to the increase in CO_2_ or immediately after the period of increased CO_2_ would be optimal to define the impacts of CO_2_ on physiological parameters. Therefore, mussels were sample during both intervals, and *n* = 7 animals were sampled immediately prior to the increase in CO_2_, whereas a second *n* = 7 animals were sampled immediately following the increase in *p*CO_2_, once *p*CO_2_ returned to control values. All samples were collected around the 12.00 h CO_2_ exposure to standardize any potential for diel variation in physiological parameters.

Haemolymph (0.5 ml for *L. cardium* and *A. plicata*; and 0.25 ml for *P. grandis*) was extracted from the anterior adductor muscle with a 1 ml syringe and 26 gauge needle (Gustafson *et al*., 2005) and then centrifuged at 12 000***g*** for 2 min. After centrifugation, the supernatant was removed, flash frozen in liquid nitrogen and stored at −80°C until processing. Mussels were sampled for haemolymph only once per sampling day, and were randomly sampled before or after the CO_2_ exposure on each sampling day over the 28 day period. On day 28 of exposure, mussels were sampled for haemolymph as stated above and then lethally sampled. Lethal mussel sampling included measurements for length, width, depth of the whole mussel using digital callipers (traceable digital carbon fiber calipers, Fisher Scientific, Pittsburg, PA, USA), and weight of the whole mussel (tissue + shell) was collected to the nearest 0.01 g using a balance (HL-300WP, A&D, Ann Arbor, MI, USA). Soft tissue dry weight (in milligrams) was determined by taking mussel soft tissues and drying them at 99°C for 24 h before weighing (Widdows *et al*., 2002). If possible, sex was determined for *L. siliquoidia* and *P. grandis* using both their external sexual dimorphism and by examination of the gills for glochidia (Trdan, 1981).

The dry weight and length of individuals within each species was not different between control and fluctuating CO_2_ treatment groups (Student's unpaired *t*-test, *P* > 0.05; Table 1). Additionally, mortalities were limited over the exposure period, but occurred for two and five *P. grandis* from the control and fluctuating *p*CO_2_ treatments, respectively, and for two *A. plicata* and one *L. cardium* exposed to the fluctuating *p*CO_2_ treatment.

**Table 1:** Results of Student's unpaired *t*-test examining the impact of dry weight and length on different *p*CO_2_ treatments

Measured variable	Species	d.f.	*t*	*P*-value
Dry weight (g)	Threeridge	17.26	−1.247	0.229
Length (cm)		21.65	0.526	0.604
Dry weight (g)	Pocketbook	22.23	0.673	0.508
Length (cm)		20.70	0.218	0.830
Dry weight (g)	Giant floater	16.82	−0.617	0.545
Length (cm)		18.95	0.350	0.730

No significant effects were detected.

### Laboratory analyses

Haemolymph Cl^−^, Mg^2+^ and Ca^2+^ concentrations were assayed in duplicate using commercially available kits (QuantiChrom assay kits Cl^−^, catalogue no. DICL-250; Mg^2+^, catalogue no. DIMG-250; Ca^2+^, catalogue no. DICA-500; BioAssay Systems, Hayward, CA, USA). Haemolymph HCO_3_^−^ and Na^+^ levels were measured by the diagnostic clinical pathology laboratory at the University of Illinois Urbana-Champaign using a Beckman chemistry analyser (Beckman Coulter AU680, Beckman Coulter, Brea, CA, USA). Quality control testing for this analyser was performed at least every 24 h. Haemolymph glucose concentrations were assayed in duplicate according to the method of Bergmeyer (1974) using a 96-well microplate and a plate spectrophotometer (Molecular Devices, SpectraMax Plus 384, Sunnyvale, CA, USA). For all assays, the inter- and intra-assay coefficients of variability were <10%.

### Statistical analyses

The effects of CO_2_ exposure on haemolymph ion levels and glucose concentrations were quantified using a two-way analysis of variance (ANOVA), with *p*CO_2_ (fluctuating or control), sampling day and their interaction (*p*CO_2_ × sampling day) entered into each model as fixed effects. Individual mussel identification number (ID), time point (i.e. sampling before or after *p*CO_2_), length, dry weight and sex (if applicable) were initially included in the models as cofactors to quantify their potential influence on response variables, but were removed because they had no significant effect on model outputs (Engqvist, 2005; Zuur *et al*., 2009). If at least one of the main effects in the ANOVA model was significant, or if the interaction term was significant, a Tukey–Kramer honestly significant difference (HSD) *post hoc* test was applied to separate means (Rohlf and Sokal, 1995). Finally, a separate Student's unpaired *t*-test was run on each species to quantify differences in dry weight and length across different *p*CO_2_ treatments.

For all statistical analyses, analysis of fitted residuals using a quantile–quantile plot (Anscombe and Tukey, 1963) was used to assess normality, while a Hartley's *F*_max_ test (Hartley, 1950), combined with visual inspection of the distribution of fitted residuals, was used to assess homogeneity of variances. If either normality or homogeneity of variance assumptions were violated (Siegel and Castellan, 1988), data were rank transformed and then re-analysed within the same parametric model described above, and the assumptions of both normality and equal variances were confirmed (Conover and Iman, 1981; Iman *et al*., 1984; Potvin and Roff, 1993). All data are presented as means ± SEM where appropriate, all tests were performed using R (version 3.2.2), and differences were considered significant if α was <0.05. For all variables and species, there was no effect of sampling before vs. after CO_2_ application (i.e. time point) for either treatment (control or fluctuating), so data from mussels sampled before and after CO_2_ application were combined.

## Results

**Figure 1:**
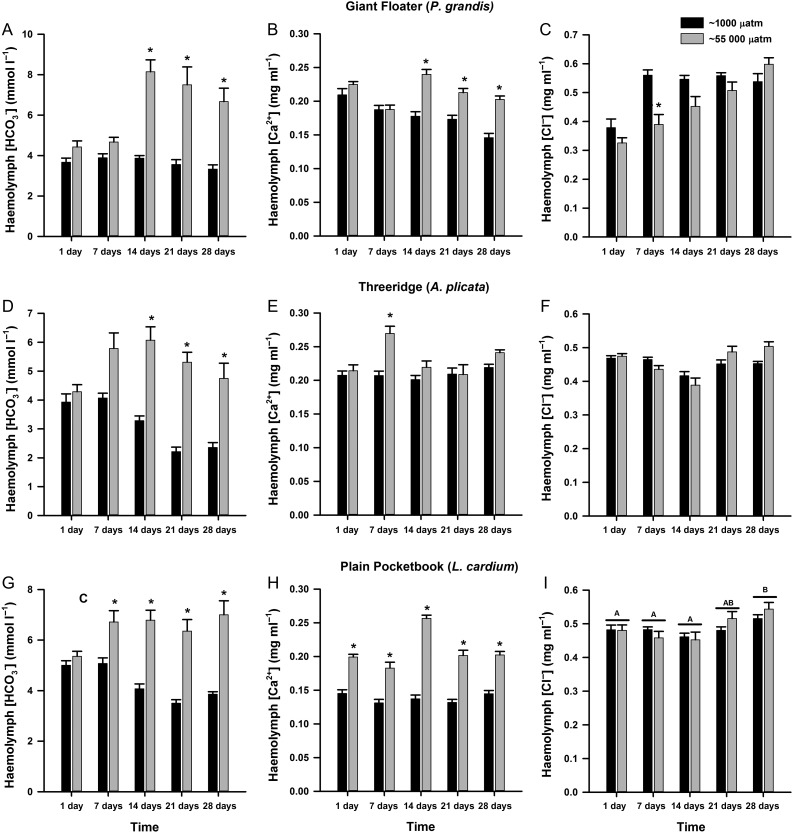
Concentrations of HCO_3_^−^, Ca^2+^ and Cl^−^ in the haemolymph of *Pyganodon grandis* (*n* = 9–14; **A–C**), *Amblema plicata* (*n* = 12–14; **D–F**) and *Lampsilis cardium* mussels (*n* = 13–14; **G–I**) exposed to two treatments of *p*CO_2_, ~1000 µatm (control) or intermittent increase at ~55 000 µatm for 1, 7, 14, 21 or 28 days. Data are presented as means + SEM. *Groups that were significantly different from the control treatment within a time point (two-way ANOVA; see Tables 2–4). For (I), there was no significant interaction between *p*CO_2_ treatment and sampling day; a bar above the treatments of a day represents a significant effect of time (two-way ANOVA).

There was a significant interaction between treatment and day for all three species of mussels for haemolymph HCO_3_^−^ (Tables 2–4). At 14 days of exposure to fluctuating *p*CO_2_, *P. grandis* (treatment × day, *F* = 13.0, *P* < 0.001; Fig. 1A) and *A. plicata* (treatment × day, *F* = 8.61, *P* < 0.001; Fig. 1D) had approximately a 2-fold increase in haemolymph HCO_3_^−^ relative to mussels held at ambient *p*CO_2_, and these concentrations remained significantly elevated for the duration of the exposure period. For *L. cardium*, haemolymph HCO_3_^−^ was significantly elevated beginning at 4 days of exposure compared with control mussels and throughout the rest of the exposure period (treatment × day, *F* = 0.52, *P* < 0.001; Fig. 1G).

**Table 2:** Results of two-way ANOVA examining the impact of fluctuating exposure to elevated *p*CO_2_ on *Pyganodon grandis* exposed to one of two different *p*CO_2_ treatments [~1000 µatm (ambient); intermittent at ~55 000 µatm] for 28 days

Measured variable	Main effects	Sum of squares	d.f.	*F*	*P*-value
HCO_3_^−^ (mmol l^−1^)	Treatment	4.84	1	115.59	**<0.001**
	Day	1.29	4	7.71	**<0.001**
	Treatment × day	2.18	4	12.99	**<0.001**
	Residuals	4.61	110		
Ca^2+^ (mg ml^−1^)	Treatment	40 708	1	61.50	**<0.001**
	Day	40 630	4	15.34	**<0.001**
	Treatment × day	22 285	4	8.42	**<0.001**
	Residuals	79 450	120		
Cl^−^ (mg ml^−1^)	Treatment	16 163	1	21.43	**<0.001**
	Day	59 308	4	19.66	**<0.001**
	Treatment × day	17 084	4	5.66	**<0.001**
	Residuals	90 518	120		
Na^+^ (g l^−1^)	Treatment	3033	1	3.87	0.0517
	Day	48 876	4	15.58	**<0.001**
	Treatment × day	9883	4	3.15	**0.0170**
	Residuals	87 826	112		
Mg^2+^ (mg ml^−1^)	Treatment	0.001	1	46.21	**<0.001**
	Day	0.002	4	15.28	**<0.001**
	Treatment × day	0.002	4	18.33	**<0.001**
	Residuals	0.003	120		
Glucose (µM)	Treatment	2656	1	2.23	0.1385
	Day	12 606	4	2.64	**0.0373**
	Treatment × day	10 084	4	2.11	0.0838
	Residuals	137 268	115		

Bold *P*-values indicate statistical significance across treatment groups within a measured variable.

**Table 3:** Results of two-way ANOVA examining the impact of fluctuating exposure to elevated *p*CO_2_ on *Amblema plicata* exposed to one of two different *p*CO_2_ treatments [~1000 µatm (ambient); intermittent at ~55 000 µatm] for 28 days

Measured variable	Main effects	Sum of squares	d.f.	*F*	*P*-value
HCO_3_^−^ (mmol l^−1^)	Treatment	8.73	1	118.33	**<0.001**
	Day	3.12	4	10.57	**<0.001**
	Treatment × day	2.54	4	8.61	**<0.001**
	Residuals	9.37	127		
Ca^2+^ (mg ml^−1^)	Treatment	0.02	1	16.06	**<0.001**
	Day	0.02	4	4.99	**<0.001**
	Treatment × day	0.02	4	4.14	**0.003**
	Residuals	0.13	127		
Cl^−^ (mg ml^−1^)	Treatment	1676	1	1.44	0.232
	Day	47 572	4	10.12	**<0.001**
	Treatment × day	16 923	4	3.63	**0.008**
	Residuals	148 094	127		
Na^+^ (g l^−1^)	Treatment	521	1	211.07	**<0.001**
	Day	26.5	4	2.68	**0.0345**
	Treatment × day	126.8	4	12.85	**<0.001**
	Residuals	313.5	127		
Mg^2+^ (mg ml^−1^)	Treatment	0.0005	1	16.41	**<0.001**
	Day	0.0015	4	11.49	**<0.001**
	Treatment × day	0.0021	4	15.68	**<0.001**
	Residuals	0.0041	125		
Glucose (µM)	Treatment	1.09	1	8.75	**0.004**
	Day	0.32	4	0.65	0.627
	Treatment × day	0.49	4	0.99	0.416
	Residuals	15.77	127		

Bold *P*-values indicate statistical significance across treatment groups within a measured variable.

**Table 4:** Results of two-way ANOVA examining the impact of fluctuating exposure to elevated *p*CO_2_ on *Lampsilis cardium* exposed to one of two different *p*CO_2_ treatments [~1000 µatm (ambient); intermittent at ~55 000 µatm] for 28 days

Measured variable	Main effects	Sum of squares	d.f.	*F*	*P*-value
HCO_3_^−^ (mmol l^−1^)	Treatment	7.28	1	141.70	**<0.001**
	Day	0.47	4	2.27	0.0653
	Treatment × day	2.10	4	0.52	**<0.001**
	Residuals	6.48	126		
Ca^2+^ (mg ml^−1^)	Treatment	0.171	1	377.5	**<0.001**
	Day	0.024	4	13.36	**<0.001**
	Treatment × day	0.022	4	12.20	**<0.001**
	Residuals	0.058	127		
Cl^−^ (mg ml^−1^)	Treatment	479	1	0.35	0.553
	Day	30 987	4	5.73	**<0.001**
	Treatment × day	4442	4	0.821	0.514
	Residuals	169 113	125		
Na^+^ (g l^−1^)	Treatment	369.3	1	134.96	**<0.001**
	Day	10.6	4	0.97	0.4273
	Treatment × day	33.4	4	3.05	**0.0194**
	Residuals	344.8	126		
Mg^2+^ (mg ml^−1^)	Treatment	0.001	1	46.24	**<0.001**
	Day	0.0003	4	2.62	**0.038**
	Treatment × day	0.0008	4	7.31	**<0.001**
	Residuals	0.004	127		
Glucose (µM)	Treatment	483	1	0.81	0.369
	Day	1938	4	0.81	0.517
	Treatment × day	2369	4	1.00	0.411
	Residuals	69 399	117		

Bold *P*-values indicate statistical significance across treatment groups within a measured variable.

A significant interaction of treatment and day was also found for all three species of mussels with respect to haemolymph Ca^2+^ concentrations(Tables 2–4). *Pyganodon grandis* had a significant elevation in haemolymph Ca^2+^ concentrations beginning at 14 days of exposure to fluctuating levels of CO_2_ relative to control mussels; however, it should be noted that control mussels also experienced a decrease in haemolymph Ca^2+^ concentrationsat 28 days compared with 1 day of treatment (treatment × day, *F* = 8.42, *P* < 0.001; Fig. 1B). For *A. plicata*, haemolymph Ca^2+^ in mussels exposed to fluctuating levels of CO_2_ was significantly elevated compared with control mussels at 7 days of exposure (treatment × day, *F* = 4.14, *P* = 0.003; Fig. 1E). A similar increase in haemolymph Ca^2+^ in *L. cardium* occurred in response to fluctuating *p*CO_2_, where levels were significantly elevated compared with control mussels for the entire period of exposure (treatment × day, *F* = 12.2, *P* < 0.001; Fig. 1H).

For *P. grandis* and *A. plicata* (Tables 2 and 3), there was a significant interaction between treatment and day for haemolymph Cl^−^ concentrations, but only a significant effect of day for *L. cardium* (Table 4). Haemolymph Cl^−^ was lower in *P. grandis* exposed to fluctuating *p*CO_2_ at 7 days of exposure relative to control mussels; however, it is important to note that this might have been attributable to a significant increase in haemolymph Cl^−^ concentrations in control mussels at 7 days compared with control mussels on day 1 of treatment (treatment × day, *F* = 5.66, *P* < 0.001; Fig. 1C). In *A. plicata*, although there was a significant interaction of treatment and day, haemolymph Cl^−^ was not significantly different in mussels exposed to fluctuating levels of CO_2_ and control mussels at any point throughout the exposure (treatment × day, *F* = 3.63, *P* = 0.008; Fig. 1F). In *L. cardium*, no significant effect of CO_2_ treatment was detected, and haemolymph Cl^−^ increased overall at 28 days of treatment (day, *F* = 5.73, *P* < 0.001; Fig. 1I).

**Figure 2:**
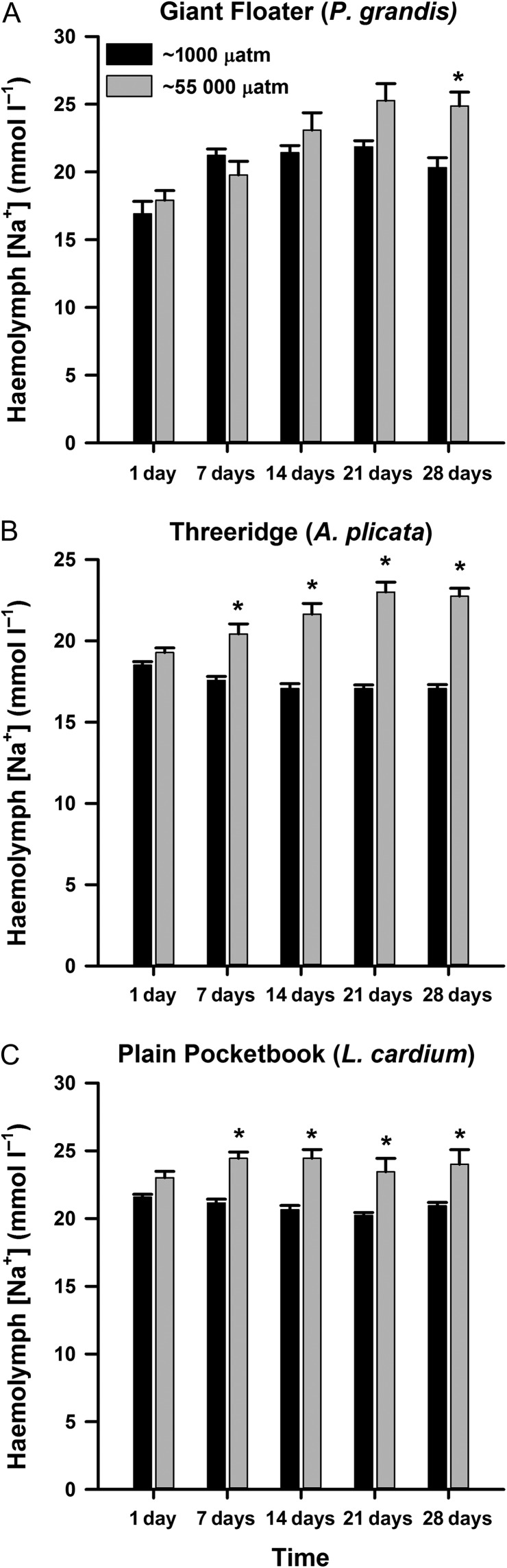
Concentrations of Na^+^ in the haemolymph of *Pyganodon grandis* (*n* = 9–14; **A**), *Amblema plicata* (*n* = 12–14; **B**) and *Lampsilis cardium* mussels (*n* = 13–14; **C**) exposed to two treatments of *p*CO_2_, ~1000 µatm (control) or intermittent increase at ~55 000 µatm for 1, 7, 14, 21 or 28 days. Data are presented as means + SEM. *Groups that were significantly different from the control treatment within a time point (two-way ANOVA; see Tables 2–4).

With respect to haemolymph Na^+^, a significant interaction between treatment and day was detected for all three species of mussels (Tables 2–4). *Pyganodon grandis* exposed to fluctuating *p*CO_2_ had a significant elevation in haemolymph Na^+^ at 28 days of exposure compared with control mussels (treatment × day, *F* = 3.15, *P* = 0.017; Fig. 2A). Haemolymph Na^+^ for both *A. plicata* (treatment × day, *F* = 12.85, *P* < 0.001; Fig. 2B) and *L. cardium* (treatment × day, *F* = 3.05, *P* = 0.0194; Fig. 2C) exposed to the fluctuating *p*CO_2_ were significantly elevated compared with control mussels beginning at 4 days and throughout the duration of the exposure period.

**Figure 3:**
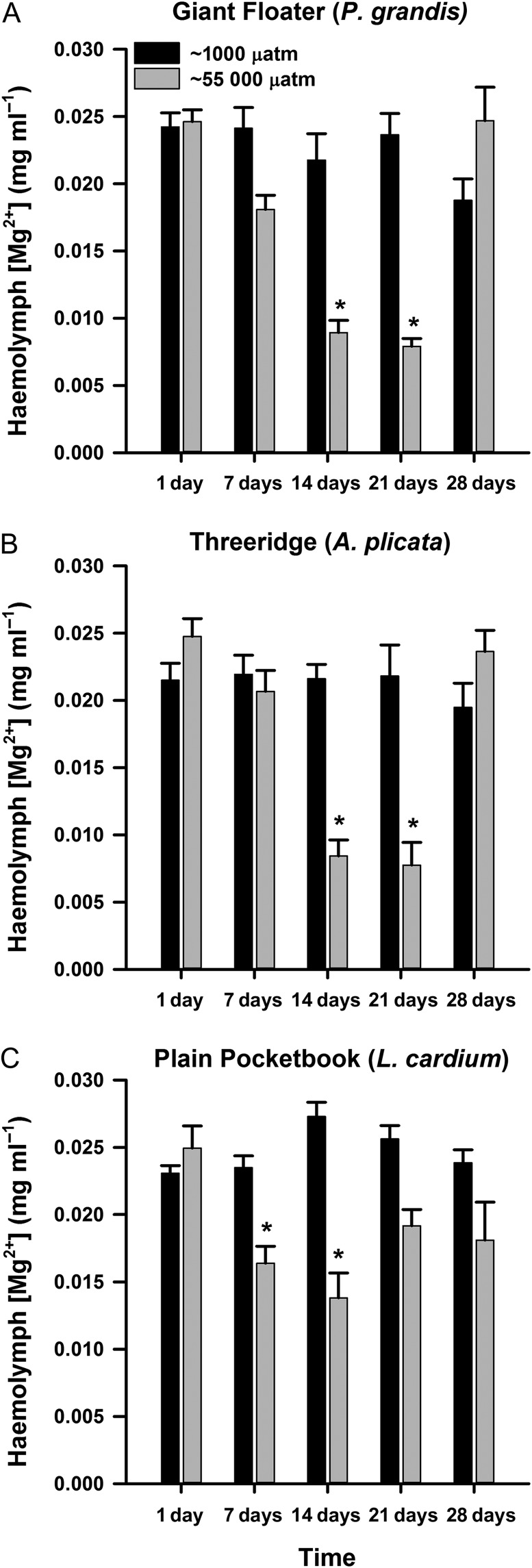
Concentrations of Mg^2+^ in the haemolymph of *Pyganodon grandis* (*n* = 9–14; **A**), *Amblema plicata* (*n* = 12–14; **B**) and *Lampsilis cardium* mussels (*n* = 13–14; **C**) exposed to two treatments of *p*CO_2_, ~1000 µatm (control) or intermittent increase at ~55 000 µatm for 1, 7, 14, 21 or 28 days. Data are presented as means + SEM. *Groups that were significantly different from the control treatment within a time point (two-way ANOVA; see Tables 2–4).

For haemolymph Mg^2+^, a significant interaction between treatment and day was also found for all three species of mussels (Tables 2–4). Haemolymph Mg^2+^ was significantly reduced at 14 and 21 days of exposure to fluctuating *p*CO_2_ compared with control mussels for *P. grandis* (treatment × day, *P. grandis*, *F* = 28.33, *P* < 0.001; Fig. 3A) and *A. plicata* (treatment × day, *F* = 15.68, *P*< 0.001; Fig. 3B), but these concentrations were no longer different from control mussels at 28 days of exposure. Likewise, haemolymph Mg^2+^ in *L. cardium* exposed to fluctuating CO_2_ levels was significantly decreased compared with control mussels on 7 and 14 days but returned to control values after 21 days of exposure (treatment × day, *F* = 7.31, *P* < 0.001; Fig. 3C).

**Figure 4:**
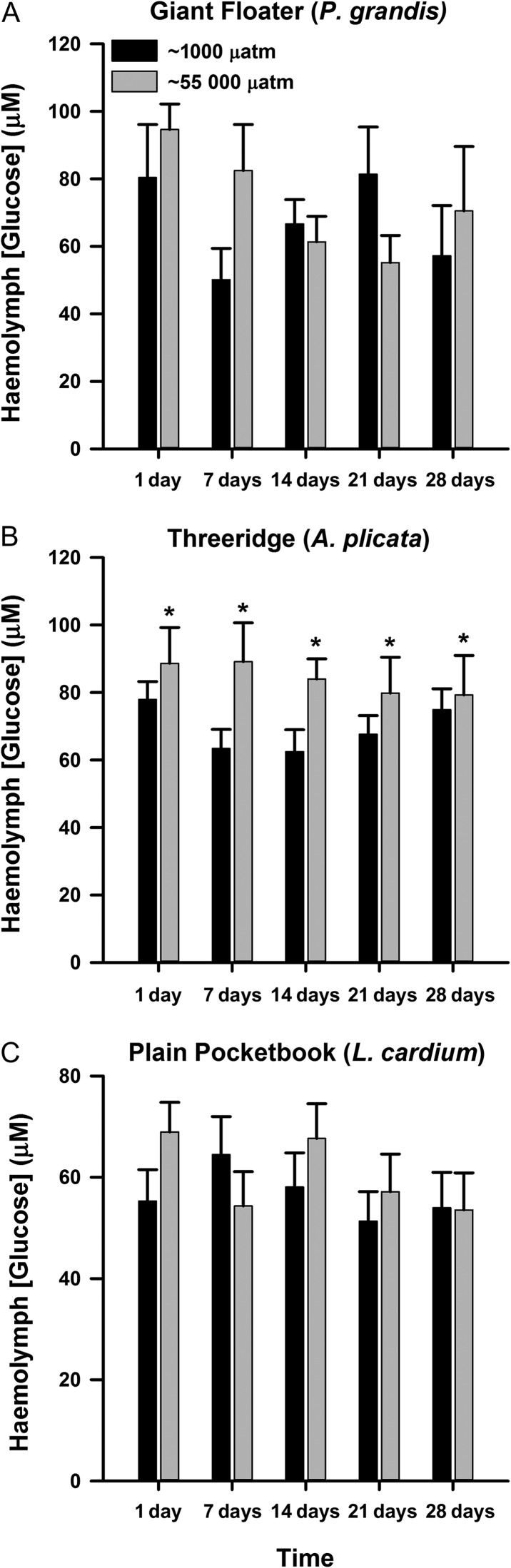
Concentrations of glucose in the haemolymph of *Pyganodon grandis* (*n* = 9–14; **A**), *Amblema plicata* (*n* = 12–14; **B**) and *Lampsilis cardium* mussels (*n* = 13–14; **C**) exposed to two treatments of *p*CO_2_, ~1000 µatm (control) or intermittent increase at ~55 000 µatm for 1, 7, 14, 21 or 28 days. Data are presented as means + SEM. For (B), there was no significant interaction between *p*CO_2_ treatment and sampling day; *significant effect of *p*CO_2_ treatment between mussels exposed to fluctuating ~55 000 µatm and those exposed to ~1000 µatm (two-way ANOVA; see Table 3).

For haemolymph glucose, there was no significant interaction of treatment and day for any species of mussel (Tables 2–4). Haemolymph glucose concentrations of *P. grandis* and *L. cardium* were unaffected by *p*CO_2_ exposure (treatment, *P* > 0.05; Fig. 4A and C). Haemolymph glucose of *A. plicata* was significantly affected by fluctuating *p*CO_2_ treatment, but not sampling day, and was elevated throughout the exposure period compared with control mussels (treatment, *F* = 8.75, *P* = 0.004; Fig. 4B).

## Discussion

Following exposure to fluctuating elevated *p*CO_2_, all three mussel species demonstrated physiological changes indicative of disturbance in acid–base regulation. Exposure to high CO_2_ often causes the acidification of internal fluids in aquatic animals (Pörtner *et al*., 2004), and one strategy for animals to buffer this internal acidosis is to increase HCO_3_^−^ concentrations (Pörtner *et al*., 2004; Pörtner, 2008). Marine mussels are thought to increase haemolymph HCO_3_^−^ by using CaCO_3_ released from the shell (Michaelidis *et al*., 2005; Bibby *et al*., 2008). In the present study, all three mussel species may have used this buffering strategy during intermittent *p*CO_2_ exposure, as both haemolymph HCO_3_^−^ and Ca^2+^ were elevated. Haemolymph HCO_3_^−^ can also be increased by reducing activity of Cl^−^–HCO_3_^−^ exchangers, thus increasing the retention of HCO_3_^−^ in the haemolymph but at the cost of Cl^−^ uptake (Byrne and Dietz, 1997). A reduction in haemolymph Cl^−^, which appears to be a short-term response to elevated *p*CO_2_ (Hannan *et al*., 2016a, b), was observed only in *P. grandis*; however, this difference may have been due to rising Cl^−^ concentrations in the haemolymph of control mussels rather than a decrease in Cl^−^ of CO_2_-treated mussels. A third strategy that mussels use to buffer acidosis is to mediate activity of Na^+^–H^+^ exchangers to increase excretion of H^+^ ions, thus also increasing Na^+^ uptake (Byrne and Dietz, 1997; Lannig *et al*., 2010; Hannan *et al*. 2016b). Increases in haemolymph Na^+^ were observed for all species of mussels exposed to fluctuating *p*CO_2_, but the timing of the elevation in haemolymph Na^+^ was species specific, as haemolymph Na^+^ concentrations for *A. plicata* and *L. cardium* mussels increased after 4 days and for *P. grandis* after 28 days of exposure. Together, the results of the present study suggest that the three species of mussels used similar mechanisms to deal with acidosis to marine mussels (i.e. manipulating haemolymph HCO_3_^−^ and H^+^ concentrations); however, species-specific differences in these responses occurred.

In addition to an acid–base disturbance, the results of our study indicate that the stress response was also activated. An indicator of stress in freshwater mussels is declining Mg^2+^ of the haemolymph, which has been associated with stressors such as elevated temperature (Fritts *et al*., 2015a), exposure to heavy metals (Hemelraad *et al*., 1990) and chronic exposures to elevated *p*CO_2_ (Hannan *et al*., 2016a, b). Haemolymph Mg^2+^ concentrations decreased by ~2-fold in all mussel species exposed to the fluctuating *p*CO_2_ treatment, but returned to control values after 28 days of exposure. In contrast, Hannan *et al*. (2016a) did not observe a return of Mg^2+^ to control values in *Fusconaia flava* exposed to ~20 000 µatm *p*CO_2_ for 32 days. In addition, *Lampsilis siliquoidea* but not *A. plicata* exposed to either 20 000 or 55 000 µatm *p*CO_2_ showed a decrease in haemolymph Mg^2+^ during 28 days of exposure, and these values returned to baseline once the CO_2_ stressor was removed (Hannan *et al*. 2016b). Although the *p*CO_2_ and the species of mussels were not the same in our study and those of Hannan *et al*. (2016a, b), these data suggest that fluctuating exposures to elevated *p*CO_2_ have a different effect on the Mg^2+^ response of unionid mussels compared with a chronic exposure. Haemolymph glucose concentrations, another indicator of stress in freshwater mussels (Patterson *et al*., 1999; Fritts *et al*., 2015a), were elevated only in *A. plicata*. Increasing glucose in response to stress comes at a cost to non-vital functions, such as growth, reproduction and movement (Patterson *et al*., 1999; Fritts *et al*., 2015a). Although the interaction of *p*CO_2_ and sampling time was not significant in the model, the elevation in glucose in *A. plicata* appeared to return to control levels following 28 days of exposure to fluctuating *p*CO_2_, suggesting that *A. plicata* recovers in terms of this stress marker by the end of the exposure period. A similar increase in haemolymph glucose was also observed for *A. plicata* exposed to a chronic elevation in *p*CO_2_ at 55 000 µatm over a 28 day period (Hannan *et al*. 2016b), suggesting that fluctuating and long-term exposure to *p*CO_2_ may have similar effects on haemolymph glucose in this species. Taken together, changes in haemolymph Mg^2+^ and glucose concentrations suggest that all three species of mussel experienced physiological stress during exposure to fluctuating *p*CO_2_; however, desensitization, acclimation or recovery might have occurred over extended exposure to the intermittent CO_2_ stressor.

Physiological changes, such as acid–base and stress responses, experienced by animals following a stressor are energetically challenging, and long-term upregulation or maintenance of these responses can lead to less energy availability for non-vital functions, such as growth and reproduction (Wendelaar Bonga, 1997). Following exposure to intermittent or repeated stressors, animals may respond to subsequent exposures in different ways (i.e. exacerbation, attenuation or no change; Reinert *et al*., 2002). Our results suggest that the duration and CO_2_ concentration used in the present study did not permit recovery between pulses of high *p*CO_2_, evidenced by the fact that mussels sampled before and after the CO_2_ exposure were not statistically different from each other. Additionally, the responses of mussels to intermittent *p*CO_2_ exposure (i.e. elevations of Ca^2+^ and Na^+^ and reduction in Mg^2+^) were similar to those observed in unionid mussels exposed to a chronically elevated *p*CO_2_ (Hannan *et al*., 2016a, b), suggesting that mussels react to the intermittent and chronic CO_2_ exposures in a similar way. However, differences in the responses of these variables during intermittent (present study) and chronic exposures (Hannan *et al*., 2016a, b) did arise during the later stages of the 28 and 32 days exposure period, respectively. For instance, as mentioned above, the concentration of Mg^2+^ returned to control values by the end of the intermittent CO_2_ exposure, whereas in previous studies using either chronic exposure to elevated CO_2_ (Hannan *et al*., 2016a, b) or elevated temperature (Fritts *et al*., 2015b), Mg^2+^ remained reduced throughout the exposure period. In addition, haemolymph Ca^2+^ (*P. grandis* and *L. cardium*) and Na^+^ (all three mussel species) remained elevated for the intermittent exposure to 55 000 µatm *p*CO_2_, whereas these ions returned to control values by 32 days of chronic exposure to ~20 000 µatm *p*CO_2_ for *F. flava* (Hannan *et al*., 2016a). This sustained increase in haemolymph Ca^2+^ and Na^+^, as well as the difference in the dynamics of the haemolymph Mg^2+^ response in at least two of the mussels species, may suggest that mussels respond differently to intermittent and chronic CO_2_ exposure. These responses also do not exclude the possibility that the differences might be species specific or driven by the difference in *p*CO_2_ used in these two studies. The present study suggests that exposure to intermittent elevations in *p*CO_2_ do result in acid–base disturbances and stress responses in unionid mussels that are both attenuated (e.g. Mg^2+^) and exacerbated (Ca^2+^ and Na^+^).

Species-specific responses observed in the present study might have resulted from a combination of differences in the physiology and behaviour of the three mussel species examined. Haemolymph Ca^2+^ was elevated in both *P. grandis* and *L. cardium* for more than half of the treatment period, whereas Ca^2+^ concentrations were elevated only on day 7 of exposure in *A. plicata*, suggesting that these species may rely differently on shell CaCO_3_ stores. In addition, a decrease in haemolymph Cl^−^ was observed only in *P. grandis* and did not occur in either *L. cardium* or *A. plicata*. These differences in the studied unionid mussels suggest that they may use different strategies to retain HCO_3_^−^ for acid–base regulation. Finally, similar elevations in haemolymph Na^+^ throughout nearly the entire *p*CO_2_ exposure period were observed in *L. cardium* and *A. plicata*, whereas haemolymph Na^+^ in *P. grandis* was elevated only at 28 days of exposure. This difference in haemolymph Na^+^ concentrations in response to *p*CO_2_ exposure suggests that *L. cardium* and *A. plicata* may rely on increased regulation of the Na^+^–H^+^ exchanger to buffer acidosis, a mechanism that may be less important for *P. grandis* until CO_2_ exposure is extended. In terms of measures of the stress response, a similar transient decrease in Mg^2+^ was observed across all species; however, haemolymph glucose was elevated only in *A. plicata*. Taken together, similar responses to intermittent elevation in *p*CO_2_ were observed across the three species examined, and the species differences that arose highlight the importance of considering multiple species when testing an organism's reaction to a stressor.

Results obtained in our study increase the understanding of responses of freshwater unionid mussels to fluctuating exposures of elevated *p*CO_2_, as modelled after a CO_2_ barrier to invasive fish movement. There is evidence that, like marine mussels, if freshwater unionid mussels are exposed to elevated *p*CO_2_ at either chronically high levels (Hannan *et al*., 2016a, b) or intermittent elevations (pres study), they will experience acid–base disturbances. If unionid mussels were to be exposed to intermittent elevations in *p*CO_2_ for an extended period of time, populations might be negatively affected owing to the increased energy demand of acid–base regulation and stress responses that may come at the expense of growth and reproduction. Additionally, resident mussel species may be affected differently, as evidenced by the observed species-specific responses to elevated *p*CO_2_, which may arise because of differences in their behaviour and physiology. It is also important to consider that fluctuating elevations in *p*CO_2_ may have similar but potentially also differential impacts compared with chronic exposures of elevated *p*CO_2_ and that, generally, exposure time and duration between applications of a stressor are important aspects to consider for study design. Taken together, the results of our study suggest that the duration and manner of *p*CO_2_ exposure (i.e. chronic vs. intermittent), as well as the species characteristics of resident unionid mussels that may be impacted, are all important factors to consider when designing, implementing and assessing the potential impacts of a CO_2_ barrier.

## Supplementary Material

Supplementary Data
