# Supplementary figures and images for: Physiological responses of three species of unionid mussels to intermittent exposure to elevated carbon dioxide
Source: Conserv Physiol. 2016 Dec 29;4(1):cow066. doi: 10.1093/conphys/cow066 (PMC5196031; doi:10.1093/conphys/cow066)

Supplementary material 1.

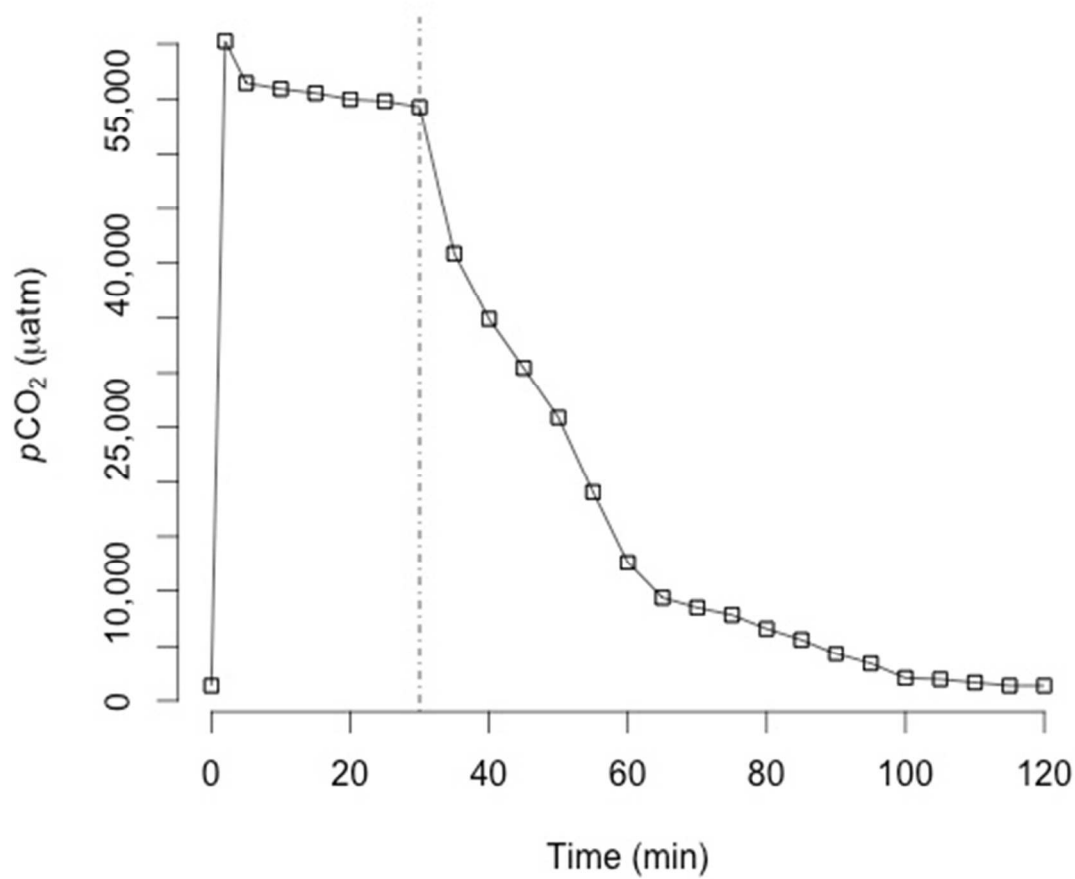

Supplement: Supplementary Data [file cow066_conphys-2016-022.pdf]
